# Deep-sea Hydrothermal Vent Bacteria as a Source of Glycosaminoglycan-Mimetic Exopolysaccharides

**DOI:** 10.3390/molecules24091703

**Published:** 2019-05-01

**Authors:** Agata Zykwinska, Laëtitia Marchand, Sandrine Bonnetot, Corinne Sinquin, Sylvia Colliec-Jouault, Christine Delbarre-Ladrat

**Affiliations:** IFREMER, Laboratoire Ecosystèmes Microbiens et Molécules Marines pour les Biotechnologies, 44311 Nantes, France; Laetitia.Marchand@ifremer.fr (L.M.); Sandrine.Bonnetot@ifremer.fr (S.B.); Corinne.Sinquin@ifremer.fr (C.S.); Sylvia.Colliec.Jouault@ifremer.fr (S.C.-J.); Christine.Delbarre.Ladrat@ifremer.fr (C.D.-L.)

**Keywords:** exopolysaccharide, glycosaminoglycan, uronic acid, hexosamine, phylogenetic analysis

## Abstract

Bacteria have developed a unique strategy to survive in extreme environmental conditions through the synthesis of an extracellular polymeric matrix conferring upon the cells a protective microenvironment. The main structural component of this complex network constitutes high-molecular weight hydrophilic macromolecules, namely exopolysaccharides (EPS). EPS composition with the presence of particular chemical features may closely be related to the specific conditions in which bacteria evolve. Deep-sea hydrothermal vent bacteria have already been shown to produce EPS rich in hexosamines and uronic acids, frequently bearing some sulfate groups. Such a particular composition ensures interesting functional properties, including biological activities mimicking those known for glycosaminoglycans (GAG). The aim of the present study was to go further into the exploration of the deep-sea hydrothermal vent IFREMER (French Research Institute for Exploitation of the Sea) collection of bacteria to discover new strains able to excrete EPS endowed with GAG-like structural features. After the screening of our whole collection containing 692 strains, 38 bacteria have been selected for EPS production at the laboratory scale. EPS-producing strains were identified according to 16S rDNA phylogeny. Chemical characterization of the obtained EPS highlighted their high chemical diversity with the presence of atypical compositional patterns. These EPS constitute potential bioactives for a number of biomedical applications, including regenerative medicines and cancer treatment.

## 1. Introduction

Extreme environments amongst deep-sea hydrothermal vents are inhabited by a large community of bacteria despite harsh conditions of temperature, pressure and the presence of toxic elements, such as sulfides and heavy metals [1]. These extremophilic bacteria are predominantly found as a biofilm, which constitutes a three-dimensional environment where cells are embedded within a self-produced matrix composed of exopolysaccharides (EPS), proteins, lipids and nucleic acids [2,3]. This extracellular polymeric matrix enables the adhesion of planktonic cells to different substrates, a key point for the further colonization of both abiotic and biotic surfaces [4]. It also plays the role of a barrier protecting cells against hostile environmental conditions, and provides nutrients for the microbial community. Several deep-sea hydrothermal vent bacteria have already been shown to synthezise extracellular polymers, mainly EPS [5,6,7]. These EPS are high-molecular weight (HMW) anionic heteropolymers constitued of osidic residues assembled in complex structures through various glycosidic linkeages. *Alteromonas*, *Pseuodoalteromonas* and *Vibrio* are the main EPS-producing species that have been isolated from deep-sea hydrothemal vents until now [5,6]. *Alteromonas* species produce highly branched EPS, among which HYD1545 [8], HYD1644 [9], HYD657 [10], ST716 [11,12] and GY785 [13,14], particularly rich in neutral sugars, mainly glucose (Glc) and galactose (Gal), and uronic acids, such as glucuronic acid (GlcA) and galacturonic acid (GalA). These EPS are also frequently substituted by organic (pyruvate, lactate) and inorganic (sulfate) acids. HYD721 EPS produced by *Pseudoalteromonas* sp. is also a slightly sulfated polysaccharide rich in neutral sugars and uronic acids [15]. In contrast to the EPS produced by *Alteromonas* and *Pseudoalteromonas* species, the HE800 EPS produced by *Vibrio diabolicus* is a linear non-sulfated polysaccharide composed only of uronic acids (GlcA) and N-acetyl hexosamines: N-acetyl-glucosamine (GlcNAc) and N-acetyl-galactosamine (GalNAc) [16,17]. A recent study reported by Delbarre-Ladrat et al. [18] put forward that general traits of the EPS composition were specific to the bacterial genera with *Alteromonas* and *Pseudoalteromonas* species mainly producing EPS rich in neutral sugars and uronic acids with some substituents, such as sulfates, and *Vibrio* species synthesizing EPS composed of uronic acids and hexosamines.

Singular structures of the deep-sea hydrothermal vent EPS with the presence of specific chemical features, including uronic acids, hexosamines and sulfate groups confer a wide range of physico-chemical and biological properties. In particular, two EPS from deep-sea hydrothermal vent bacteria have been shown to exhibit interesting biological activities close to glycosaminoglycans (GAG), such as heparin and hyaluronic acid (HA). Indeed, GAG-like properties of the native HMW GY785 EPS and its low-molecular weight (LMW) highly sulfated derivatives have been demonstrated in tissue engineering of cartilage [19,20]. Anticoagulant [21] and antimetastatic [22] properties of LMW GY785 derivatives were also reported. HE800 EPS, presenting some structural similarities to HA, displayed instead biological activities in bone [23] and skin [24] regeneration. It becomes therefore that extremophilic marine bacteria constitute a source of molecules endowed with diverse biological properties that can be explored for several biomedical applications. A wide bacterial biodiversity ensures a large chemical diversity of the molecules that can be obtained. In addition, the production of these EPS by fermentation allows a renewable and reproducible recovery of polysaccharides with conserved compositions and structures. Moreover, since polysaccharides are produced by bacteria directly into the culture medium, no chemical extraction is required to recover them, in contrast to the polysaccharides obtained from plant and animal tissues. 

In the present study, the exploration of a large collection of bacteria isolated from deep-sea hydrothermal vents was undertaken in order to discover new EPS with particular GAG-like composition, i.e., rich in uronic acids, hexosamines and sulfates. Initial screening on 692 strains led to selection of 38 bacteria potentially able to secrete EPS of desired composition. Polysaccharide production carried out at a laboratory scale resulted in the recovery of 31 HMW anionic EPS, most of them of unusual compositional patterns, when compared to two laboratory references, namely GY785 and HE800 EPS [13,16].

## 2. Results

### 2.1. Screening for EPS-Producing Bacteria

The screening for bacteria able to synthesize EPS of a particular chemical composition mimicking that of GAG from the deep-sea hydrothermal vent IFREMER (French Research Institute for Exploitation of the Sea) collection was first performed. To rapidly assess the presence of negatively charged polymers, the 692 supernatants recovered in 1 mL cultures were analyzed by electrophoresis on agarose gel. Indeed, the electrophoretic migration is allowed by the charge of molecules, while subsequent staining of gels with Stains-all cationic dye evidences anionic compounds such as polymers [25,26] (Figure S1). HMW negatively charged GY785 and HE800 EPS were used as references. Differences in the coloration observed suggested that EPS of diverse chemical compositions were secreted, as described by Andrade et al. [27] for GAG identification. Subsequently, based on electrophoretic profiles, optical density measured for each strain after 48 h of incubation, and by taking into account the environment from which strains were isolated, including rocks, sediments, diluted vent fluids or seawater columns as well as various organisms, such as worms, shrimps, mussels or invertebrates, 38 strains were selected for EPS production at laboratory scale (Table S1).

### 2.2. EPS Production at Laboratory Scale: Chemical and Physico-Chemical Characterization

Among 38 strains selected for EPS production at laboratory scale, seven strains secreted only limited amounts of EPS (Table S1). Therefore, further chemical and physico-chemical analyses were carried out on 31 EPS recovered with higher yields. In parallel, identification of the EPS-producing strains was performed based on 16S rRNA gene analyses.

**Figure 1:**
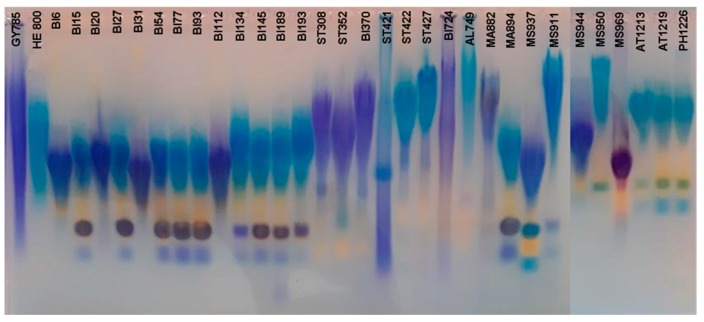
Electrophoresis on agarose gel with Stains All staining of EPS produced by 31 deep-sea hydrothermal vent bacteria.

Broad extended smears characteristic of polydisperse HMW negatively charged polymers, such as GY785 and HE800 EPS, were observed after electrophoresis on agarose gel using Stains All for all the EPS produced (Figure 1). The differences in coloration resulted most likely from the differences in chemical composition between the EPS, as observed during the screening step (Figure S1). Small anionic molecules observed in lower parts of the gel indicated that other components, such as proteins and/or other glycopolymers were also co-extracted with the EPS from the culture supernatants. The presence of nucleic acids in high amounts was excluded by Sybr safe revealing (data not shown).

Recovery yield, chemical composition, i.e., monosaccharide, protein and sulfur contents as well as the weight-average molecular weight and the polydispersity index of the EPS obtained were determined, and compared to GY785 and HE800 EPS (Table S2). In the same culture conditions, bacteria produced EPS with different yields, ranging from the lowest yield of 0.12 g/L for MS882 to the highest yield of 1.25 g/L reached for BI370. This was consistent with the production yields usually observed for marine bacteria [18]. The weight-average molecular weight ranged from 1,400,000 g/mol for BI134 to 5,100,000 g/mol for MA882, confirming the production of HMW EPS, observed by electrophoresis on agarose gel (Figure 1). The polydispersity index, Ip, was close to 1, which indicated a high monodispersity of the EPS produced. The protein content within the majority of the EPS was low, ranging from 1 wt% for ST422 to 7 wt% for AT1219, except for ST421 (12 wt%), MS950 (16 wt%) and MS969 (13 wt%). The presence of proteins was most likely due to their co-extraction during EPS purification from the culture supernatants. Sulfur was detected only in two EPS, namely MS969 and ST421, which were slightly sulfated, as their contained 2 wt%S and 3 wt%S, respectively (Table S2).

**Figure 2:**
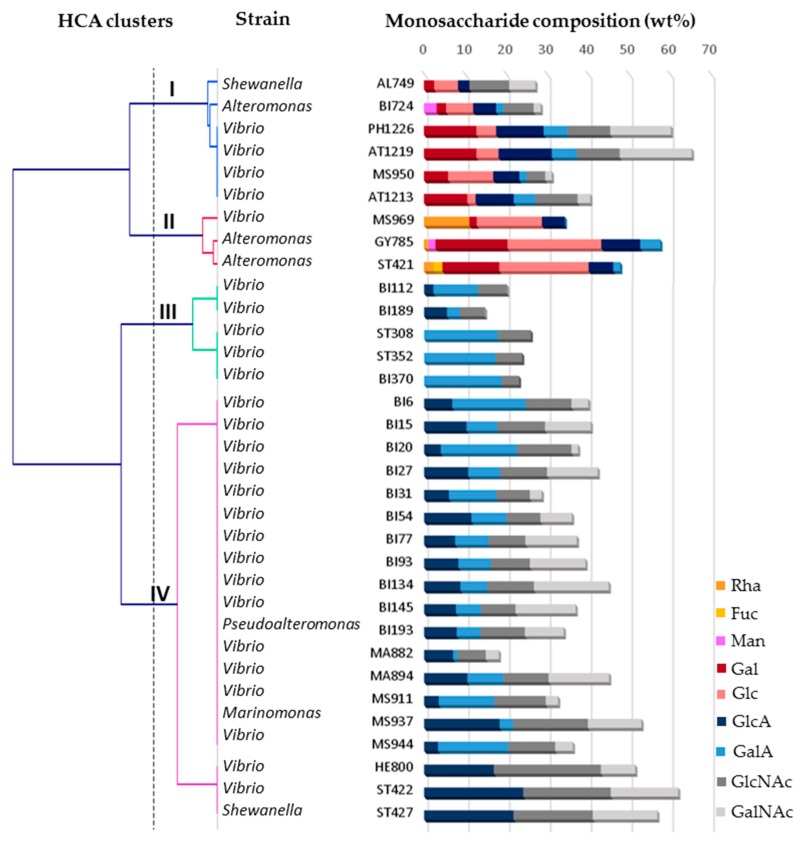
HCA clusters, bacterial strain identification and monosaccharide composition (wt%) of the EPS produced at laboratory scale. GY785 EPS and HE800 EPS were used as references.

Hierarchical cluster analysis (HCA), aiming to group subjects with similar features into clusters, was applied to further analyse the data. HCA performed on three variables, namely monosaccharide composition, protein and sulfur contents allowed to evidence four main clusters at 90% of dissimilarity (Figure 2).

Cluster I contained six EPS displaying unusual osidic composition. They contained neutral sugars, mainly Gal and Glc, and uronic acids with the presence of both GlcA and GalA, except for AL749, only composed of GlcA. Both hexosamines, i.e., GlcNAc and GalNAc were also quantified in high amounts in all the six EPS. Cluster I EPS were mainly produced by Vibrio species with one Shewanella and one Alteromonas species. Three EPS belonging to cluster II were particularly rich in neutral sugars, such as rhamnose (Rha) and Glc for MS969, and Gal and Glc for ST421, and contained low amounts of uronic acids. These two EPS were devoid of hexosamine residues. Osidic composition of ST421 EPS was similar to GY785 EPS, and both polysaccharides were produced by *Alteromonas* species, while MS969 was secreted by *Vibrio* species. The cluster II also encompasses the sulfated EPS. Cluster III contained five EPS exclusivelly produced by *Vibrio* species, which were composed of GlcNAc residues and uronic acids. Both GlcA and GalA were present in BI112 and BI189 EPS, while only GalA was detected in ST308, ST352 and BI370. Remaining EPS were identified in cluster IV. They were composed of both hexosamines, GlcNAc and GalNAc as well as uronic acids, GlcA and GalA, excepting ST422 and ST427 EPS, which contained only GlcA residues. The monosaccharide composition of these two latter EPS was close to HE800 EPS composition. In cluster IV, a majority of the EPS was produced by *Vibrio* species and only three other species, *Pseudoalteromonas*, *Marinomonas* and *Shewanella*, were identified. 

## 3. Discussion

Previous studies carried out on deep-sea hydrothermal vent bacteria, recovered during diverse oceanographic cruises, led to the discovery of EPS endowed with physico-chemical and biological, in particular GAG-like, properties [19,21,22,24,28]. GAG are anionic polysaccharides constituted of a disaccharide repeating unit containing uronic acid (GlcA or iduronic acid, IdoA) or neutral sugar (Gal) and hexosamine (glucosamine, GlcN or galactosamine, GalN). GAG are characterized by their high structural heterogeneity, arising not only from the composition of the building block and the type of glycosidic linkage between the residues, but also from the fact that these residues may be substituted with an acetyl and/or sulfate group at different positions [29]. Due to their anionic nature, GAG bind various signaling proteins (chemokines and growth factors) and are thus involved in both physiological and pathological processes, including organogenesis, wound healing, inflammation, coagulation, thrombosis, cancer growth and metastasis [30,31,32,33]. In consequence, GAG are widely explored for their therapeutic potential. However, their high structural heterogeneity considerably limits their biomedical applications. In addition, the animal origin of GAG, except for HA currently produced by *Streptococcus equi* subsp. *zooepidemicus* [34], implies complex extraction and purification, as well as the possibility of contamination by prions and viruses. Therefore the research of GAG-like molecules from alternative sources is under intense development. 

**Figure 3:**
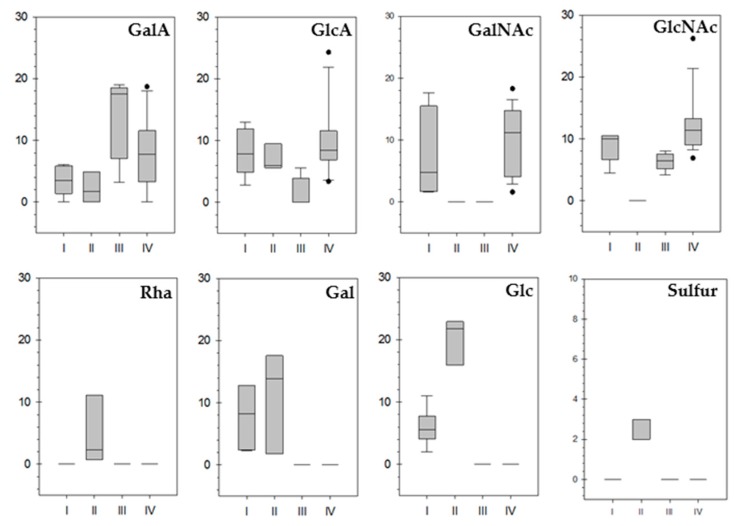
Box plot representation of the EPS chemical composition as a function of HCA clusters. In the boxes, the 25th, 50th and 75th percentiles are indicated by the bottom, middle and top lines, respectively. Whiskers show the 10th and 90th percentiles. Individual dots are the outliers.

The valuable properties of the deep-sea hydrothermal vent EPS mimicking those of GAG from animal tissues, especially in the case of GY785 and HE800 EPS, result from their original structures and particular chemical compositions. The aim of the present study was to further explore the deep-sea hydrothermal vent IFREMER collection of bacteria to discover new strains able to produce GAG-like EPS, i.e., rich in uronic acid and hexosamine residues, and additionally bearing sulfate groups. To rapidly assess the presence of HMW negatively charged polymers among the bacteria screened, electrophoresis on agarose gel was applied and revealed with Stains All. Selected EPS-producing strains were then grown at laboratory scale. EPS obtained differed by their osidic composition and sulfur content. HCA analysis revealed that four clusters can be distinguished based on EPS chemical composition. EPS rich in uronic acids were present in all four clusters (Figure 3). EPS with high GalA amounts were found in cluster III and IV, and those rich in GlcA were mainly present in cluster IV. EPS contained also hexosamines with important amounts of GalNAc quantified in clusters I and IV, while GlcNAc residues were only absent in cluster II. EPS rich in neutral sugars, such as Rha, Gal and Glc were identified in clusters I and II. Rha was only detected in cluster II EPS. EPS substituted with sulfur groups were only present in cluster II.

The present study puts forward the wide chemical diversity of EPS that deep-sea hydrothermal vent bacteria are able to synthesize. This is for the first time that the EPS rich in uronic acids, hexosamines and neutral sugars are identified within this collection (cluster I). Interestingly, they were mainly produced by *Vibrio* species, which have previously been shown to predominantly synthesize EPS rich in uronic acids and hexosamines [13,16,18]. Only a few other bacteria from marine environments have been described to secrete EPS of such osidic composition. *Pseudoalteromonas* sp. strain TG12 synthesized an EPS rich in Glc, xylose (Xyl), mannose (Man), GalA, GlcA and GlcNAc [35], while *Vibrio alginolyticus* (CNCM I-4151) excreted an EPS composed of Gal, GlcA, GalA and GlcNAc [36]. Glc, GalA, GlcA, GalNAc and GlcNAc were also the main constituents of the EPS secreted by a marine bacterium, *Vibrio neocaledonicus* [37]. Among deep-sea hydrothermal vent EPS produced in the present study, two of them, namely MS969 and ST421 EPS (cluster II) were mainly composed of neutral sugars with low amounts of uronic acids. EPS were devoid of hexosamines and were the sole EPS bearing sulfate groups (Figure 2 and Figure 3). MS969 EPS contained high amounts of Rha residue. The presence of this rare sugar responsible for some biological activities was also reported in the EPS secreted by *Pseudoalteromonas* sp. strains isolated from ocean sediments [38] and Antarctic sea water [39]. A marine bacterium, *Alteromonas* sp. JL2810 was also shown to synthesize EPS constituted of Rha, Man and GalA [40]. ST421 EPS chemical composition was very close to that of GY785 EPS and both EPS were produced by *Alteromonas* species [13,14]. Atypical osidic composition was observed for three other EPS excreted by *Vibrio* species, namely ST308, ST352 and BI370 (cluster III), constituted only of GalA and GlcNAc. To date, only one marine strain, *Vibrio alginolyticus* (CNCM I-4994), has been described to synthesize an EPS composed of these two residues [41]. In comparison, a disaccharide repeating unit of a well-known GAG, HA, is composed of GlcA and GlcNAc [42]. However, the majority of the EPS produced (cluster IV) were rich both in 2 types of uronic acids and two types of hexosamine, which was observed for the first time within this collection. A similar osidic composition was recently reported for a capsular EPS surrounding cells of the psychrophilic marine bacterium, *Colwellia psychrerythraea* 34H, which contained GlcA, GalA, GlcNAc and GalNac residues in its structure [43]. Finally, within cluster IV, two EPS, namely ST422 and ST427 EPS displayed the same osidic composition that HE800 EPS [16,17]. 

The majority of the selected bacteria was *Vibrio* sp. The relationship between EPS osidic composition and phylogenetic position of the producing strain is not as clear as previously described [18]. In our present work, the specific traits in EPS composition previously identified at the genus level are not confirmed since *Alteromonas* sp. BI724 produces an EPS with NAc-hexosamines and the EPS of *Vibrio* sp. MS969 is sulfated. This is the first description of a sulfated EPS produced by a *Vibrio* strain. In addition, the *Pseudoalteromonas* sp. BI193 EPS is not sulfated. These new results can be explained by the larger biodiversity screened in this study. In addition, in our previous work, bacteria that were mucoid on agar medium plates were preselected. Nevertheless, all bacteria are Gram-negative, gamma-proteobacteria strains belonging to three different orders, i.e., Vibrionales for *Vibrio* sp., Oceanospirillales for *Marinomonas* sp. and Alteromonadales for the three genera *Shewanella*, *Alteromonas* and *Pseudoalteromonas*.

It therefore becomes apparent from this study that deep-sea hydrothermal vent bacteria can be considered as an inexhaustible source of EPS displaying various compositional patterns. The capacity to produce EPS of particular composition was most likely a necessity for bacteria to survive and proliferate in such an extreme environment. Especially, EPS rich in uronic acids may bind different metals, such as Cd^2+^, Pb^2+^, Ni^2+^, Al^3+^, Cu^2+^, thus allowing to reduce exposure of cells to excessive concentrations of these heavy and toxic elements [35,40,44]. Cation-binding properties of EPS lead also to the formation of gels through the cross-linking between polysaccharide chains and divalent cations, e.g., Ca^2+^, abundantly present in seawater. Such a gelled microenvironment remains crucial for the biofilm setting [45]. Nowadays, the ability of bacteria to produce EPS of such a particular composition, conferring different functional properties, can be exploited for a number of biomedical applications, including cancer treatment and tissue regeneration. Further work will consist of a determination of the fine structure of some unusual EPS characterized in this study, required for an understanding of the structure-function relationship. In particular, the structure of AT1219 EPS from cluster I, rich in neutral sugars (Glc, Gal), uronic acids (GlcA, GalA) and hexosamines (GlcNAc, GalNAc) and as well as BI370 from cluster III, containing only GalA and GlcNAc will firstly be assessed.

## 4. Materials and Methods 

### 4.1. Deep-Sea Hydrothermal Vent IFREMER Collection of Bacteria

Strains (n = 692) were isolated from samples collected during the IFREMER oceanographic cruises organized to explore the deep-sea hydrothermal vent biodiversity: MMVT (AL), Atos (AT), Biolau (BI), Guaynaut (GY), Hero (HE), Hydronaut (HY), MAR (MA), Microsmoke (MS), Phare (PH), Starmer (ST) and Marvel (MV). Sample names started with the two letters of the cruise, followed by the isolate number.

### 4.2. Screening for EPS-Producing Bacteria

Bacteria were grown in Zobell-like medium composed of aquarium salts (33.3 g/L), ultrafiltrated yeast extract (1 g/L) and tryptone (4 g/L). The medium was buffered with HEPES (50 mM) and PIPES (50 mM), and pH was adjusted to 7.5. Screening of bacteria was performed in 1.2 mL tubes by incubating 100 µL of inoculum with 900 µL of Zobell-like medium supplemented with glucose (30 g/L) at 30 °C under continuous agitation (150 rpm). After 48h of incubation, bacterial growth was assessed by measurement of absorbance at 620 nm. Tubes were then centrifuged for 15 min, 12,000 g at 20 °C. Supernatants were kept at −20 °C before electrophoresis on agarose gel, while pellets were stored at −80 °C for 16S rRNA analysis. 

16S rDNA genes were amplified by polymerase chain reaction (PCR) on pellets recovered after the screening step. Pellets were suspended in 320 µL of sterile water and diluted 10-fold before analysis. PCR were carried out, as previously described [18], with GoTaq ColorlessMasterMix 2x (Promega) at 55 °C hybridization and 1 min elongation using appropriate primers (8F (5’-AGAGTTTGATCATGGCTCAG-3’) and 1489R (5’- GTTACCTTGTTACGACTTCAC-3’)). Amplicons were analyzed on 1% agarose gel electrophoresis with Sybr safe (Invitrogen) and purified from gel with the GeneJET Gel Extraction and DNA Cleanup Micro Kit (Thermofisher) before sequencing (GATC Biotech). Bioedit software was applied to build a consensus sequence using forward and reverse sequences [46] (Hall, 1999). Strain identification was performed by nucleotide Blast (megablast program) on NCBI nr/nt Nucleotide Collection (https://www.ncbi.nlm.nih.gov/).

### 4.3. EPS Production at Laboratory Scale

EPS production of selected bacterial strains was carried out in Zobell-like medium (400 mL) supplemented with glucose in a 1 L Erlenmeyer incubated at 30 °C, 150 rpm for 48 h. Each culture was then centrifuged for 45 min, 8000 g at 20 °C. Supernatants were filtered on 2.6 µm glass microfiber membranes (Whatman, Maidstone, UK), purified by tangential ultrafiltration on a 100 kDa cut-off membrane (Millipore, VWR, Fontenay-sous-Bois, France) and freeze-dried prior to analysis.

### 4.4. EPS Characterization

#### 4.4.1. Electrophoresis on Agarose Gel

Agarose gel (0.7 % w/v) was prepared in TAE buffer (0.04 M Tris-acetate; 0.01 M EDTA, pH 8.5), as previously described [26]. 30 µL of EPS samples in native sample buffer were loaded on gel wells and electrophoresis equipped with a cooling system was run in TAE buffer for 2h. Gel was then fixed in 25% (v/v) isopropanol for 4 h, before being colored overnight in the dark by the cationic carbocyanine dye Stains All (1-Ethyl-2-[3-(1-ethylnaphto [1,2-d]thiazoline-2-ylidene)-2-methyl-propenyl]naphto[1,2-d]thiazolium bromide); solution was prepared at 0.005% in dimethylformamide, isopropanol and 300 mM Tris HCl pH8 [47]. Gels were then destained for 2 h in water under natural light.

#### 4.4.2. Osidic Composition

Monosaccharide composition was determined according to Kamerling et al. [48] method, modified by Montreuil et al. [49]. EPS produced were firstly hydrolyzed for 4 h at 100 °C in MeOH/HCl 3N (Merck). The obtained methyl glycosides were then converted to trimethylsilyl derivatives using *N*,*O*-bis(trimethylsilyl)trifluoroacetamide and trimethylchlorosilane (BSTFA:TMCS) 99:1 (Merck). Quantification of the per-*O*-trimethylsilyl methyl glycosides formed was performed using gas chromatography (GC-FID, Agilent Technologies 6890N, Santa Clara, CA, USA). *Myo*-inositol was used as internal standard.

#### 4.4.3. Protein Content

The protein content in the EPS samples was determined using bicinchoninic acid method (BCA-Kit, Sigma).

#### 4.4.4. Sulfate Content

The linked ester sulfate groups were quantified by High-Performance Anion-Exchange Chromatography (HPAEC) using Dionex DX-500 ion chromatographic instrument controlled with Chromeleon^®^ software (Dionex, Sunnyvale, CA, USA), as previously described by Chopin et al. [50].

#### 4.4.5. Molecular Weight Analysis

The weight-average molecular weight was determined by High-Performance Size-Exclusion Chromatography (HPSEC, Prominence Shimadzu Co, Kyoto, Japan) coupled with a multiangle light scattering (MALS, Dawn Heleos-II, Wyatt Technology, Santa Barbara, CA, USA) and a differential refractive index (RI) (Optilab Wyatt technology, Santa Barbara, CA, USA) detectors. The molecular weight was calculated using a refractive index increment characteristic of polysaccharides, *dn/dc* = 0.145 mL/g.

### 4.5. Hierarchical Cluster Analysis (HCA)

EPS were statistically clustered on the basis of their chemical composition (monosaccharide, protein and sulfate contents). A binary matrix was constructed on the presence (=1) or absence (=0) of monosaccharides, sulfur and protein contents. Analysis was carried out with Dice’s coefficient. Ward’s method was used for the classification. HCA was performed using XLSTAT 2015.5 software (Addinsoft, Paris, France).

## Supplementary Materials

The following are available online: Figure S1: Example of electrophoretic analysis on agarose gel with Stains All staining performed on culture broth supernatants recovered after 48 h of incubation of 21 deep-sea hydrothermal vent strains in Zobell medium supplemented with glucose during the screening step. GY785 EPS and HE800 EPS were used as references. Table S1: Deep-sea hydrothermal vent strains selected for EPS production at laboratory scale. Table S2: Recovery yield, weight-average molecular weight (Mw), polydispersity index (Ip) and chemical composition (protein, sulfur and monosaccharide contents) of 31 EPS produced at laboratory scale. GY785 EPS and HE800 EPS were used as references.

## References

[1] Jannasch H.W., Taylor C.D. (1984). Deep-sea microbiology. Annu. Rev. Microbiol..

[2] Wingender J., Neu T., Flemming H.-C. (1999). Microbial Extracellular Polymeric Substances.

[3] Sutherland I.W. (2001). Biofilm exopolysaccharides: A strong and sticky framework. Microbiology.

[4] Flemming H.-C., Wingender J. (2010). The biofilm matrix. Nature Rev..

[5] Guezennec J. (2002). Deep-sea hydrothermal vents: A new source of innovative bacterial exopolysaccharides of biotechnological interest?. J. Ind. Microbiol. Biotechnol..

[6] Delbarre-Ladrat C., Sinquin C., Lebellenger L., Zykwinska A., Colliec-Jouault S. (2014). Exopolysaccharides produced by marine bacteria and their applications as glycosaminoglycan-like molecules. Front. Chem..

[7] Casillo A., Lanzetta R., Parrilli M., Corsaro M.M. (2018). Exopolysaccharides from marine and marine extremophilic bacteria: Structures, properties, exological roles and applications. Mar. Drugs.

[8] Vincent P., Pignet P., Talmont F., Bozzi L., Fournet B., Guezennec J. (1994). Production and characterization of an exopolysaccharide excreted by a deep-sea hydrothermal vent bacterium isolated from the polychaete annelid *Alvinella pompejana*. Appl. Environ. Microbiol..

[9] Dubreucq G., Domon B., Fournet B. (1996). Structure determination of a novel uronic acid residue isolated from the exopolysaccharide produced by a bacterium originating from deep sea hydrothermal vents. Carbohydr. Res..

[10] Cambon-Bonavita M.A., Raguenes G., Jean J., Vincent P., Guézennes J. (2002). A novel polymer produced by a bacterium isolated from a deep-sea hydrothermal vent polychaete annelid. J. Appl. Microbiol..

[11] Raguénès G., Pignet P., Gauthier G., Peres A., Christen R., Rougeaux H., Barbier G., Guezennec J. (1996). Description of a new polymer-secreting bacterium from a deep-sea hydrothermal vent, *Alteromonas macleodii* subsp. fijiensis, and preliminary characterization of the polymer. Appl. Environ. Microbiol..

[12] Rougeaux H., Talaga P., Carlson R.W., Guezennec J. (1998). Structural studies of an exopolysaccharide produced by *Alteromonas macleodii* subsp. fijiensis originating from a deep-sea hydrothermal vent. Carbohydr. Res..

[13] Raguénès G.H., Peres A., Ruimy R., Pignet P., Christen R., Loaëc M., Rougeaux H., Barbier G., Guezennec J. (1997). *Alteromonas infernus* sp. nov., a new polysaccharide-producing bacterium isolated from a deep-sea hydrothermal vent. J. Appl. Microbiol..

[14] Roger O., Kervarec N., Ratiskol J., Colliec-Jouault S., Chevolot L. (2004). Structural studies of the main exopolysaccharide produced by the deep-sea bacterium *Alteromonas infernus*. Carbohydr. Res..

[15] Rougeaux H., Guezennec J., Carlson R.W., Kervarec N., Pichon R., Talaga P. (1999). Structural determination of the exopolysaccharide of *Pseudoalteromonas* strain HYD 721 isolated from a deep-sea hydrothermal vent. Carbohydr. Res..

[16] Raguénès G., Christen R., Guezennec J., Pignet P., Barbier G. (1997). *Vibrio diabolicus* sp. nov., a new polysaccharide-secreting organism isolated from a deep-sea hydrothermal vent polychaete annelid, *Alvinella pompejana*. Int. J. Syst. Bact..

[17] Rougeaux H., Kervarec N., Pichon R., Guezennec J. (1999). Structure of the exopolysaccharide of *Vibrio diabolicus* isolated from a deep-sea hydrothermal vent. Carbohydr. Res..

[18] Delbarre-Ladrat C., Leyva Salas M., Sinquin C., Zykwinska A., Colliec-Jouault S. (2017). Bioprospecting for exopolysaccharides from deep-sea hydrothermal vent bacteria: Relationship between bacterial diversity and chemical diversity. Microorganisms.

[19] Merceron C., Portron S., Vignes-Colombeix C., Rederstorff E., Masson M., Lesoeur J., Sourice S., Sinquin C., Colliec-Jouault S., Weiss P. (2012). Pharmacological modulation of human mesenchymal stem cell chondrogenesis by a chemically over-sulphated polysaccharide of marine origin: Potential application to cartilage regenerative medicine. Stem Cells.

[20] Rederstorff E., Rethore G., Weiss P., Sourice S., Beck-Cormier S., Mathieu E., Maillasson M., Jacques Y., Colliec-Jouault S., Fellah B.H. (2017). Enriching a cellulose hydrogel with a biologically active marine exopolysaccharide for cell-based cartilage engineering. J. Tissue Eng. Regen. Med..

[21] Colliec-Jouault S., Chevolot L., Helley D., Ratiskol J., Bros A., Sinquin C., Roger O., Fischer A.-M. (2001). Characterization, chemical modifcations and in vitro anticoagulant properties of an exopolysaccharide produced by *Alteromonas infernus*. Biochim. Biophys. Acta.

[22] Heymann D., Ruiz-Velasco C., Chesneau J., Ratiskol J., Sinquin C., Colliec-Jouault S. (2016). Anti-metastatic properties of a marine bacterial exopolysaccharide-based derivative designed to mimic glycosaminoglycans. Molecules.

[23] Zanchetta P., Lagarde N., Guezennec J. (2003). A new bone-healing material: A hyaluronic acid-like bacterial exopolysaccharide. Calcif. Tissue Int..

[24] Senni K., Gueniche F., Changotade S., Septier D., Sinquin C., Ratiskol J., Lutomski D., Godeau G., Guezennec J., Colliec-Jouault S. (2013). Unusual glycosaminoglycans from a deep sea hydrothermal bacterium improve fibrillar collagen structuring and fibroblast activities in engineered connective tissues. Mar. Drugs.

[25] Rigouin C., Delbarre-Ladrat C., Sinquin C., Colliec-Jouault S., Dion M. (2009). Assessment of biochemical methods to detect enzymatic depolymerization of polysaccharides. Carbohydr. Polym..

[26] Zykwinska A., Tripon-Le Berre L., Sinquin C., Ropartz D., Rogniaux H., Colliec-Jouault S., Delbarre-Ladrat C. (2018). Enzymatic depolymerization of the GY785 exopolysaccharide produced by the deep-sea hydrothermal bacterium *Alteromonas infernus*: Structural study and enzyme activity assessment. Carbohydr. Polym..

[27] Andrade J.P.S., Oliveira C.P., Tovar A.M.F., Mourão P.A.S., Vilanova E. (2018). A color-code for glycosaminoglycans identification by means of polyacrylamide gel electrophoresis stained with the cationic carbocyanine dye Stains-all. Electrophoresis.

[28] Zykwinska A., Marquis M., Godin M., Marchand L., Sinquin C., Garnier C., Jonchère C., Chédeville C., Le Visage C., Guicheux J. (2019). Microcarriers based on glycosaminoglycan-like marine exopolysaccharide for TGF-β1 long-term protection. Mar. Drugs.

[29] Gandhi N.S., Mancera R.L. (2008). The structure of glycosaminoglycans and their interactions with proteins. Chem. Biol. Drug Des..

[30] Bourin M.C., Lindahl U. (1993). Glycosaminoglycans and the regulation of blood coagulation. Biochem. J..

[31] Afratis N., Gialeli C., Nikitovic D., Tsegenidis T., Karousou E., Theocharis A.D., Pavão M.S., Tzanakakis G.N., Karamanos N.K. (2012). Glycosaminoglycans: Key players in cancer cell biology and treatment. FEBS J..

[32] Salbach J., Rachner T., Rauner M., Hempel U., Anderegg U., Franz S., Simon J.-C., Hofbauer L. (2012). Regenerative potential of glycosaminoglycans for skin and bone. J. Mol. Med..

[33] Kovensky J., Grand E., Uhrig M.L., Goyanes S.N., D’Accorso N.B. (2017). Applications of glycosaminoglycans in the medical, veterinary, pharmaceutical, and cosmetic fields. Industrial Applications of Renewable Biomass Products: Past, Present and Future.

[34] Rehm B.H.A. (2009). Microbial Production of Biopolymers and Polymer Precursors: Applications and Perspectives.

[35] Gutierrez T., Shimmield T., Haidon C., Black K., Green D.H. (2008). Emulsifying and metal ion binding activity of a glycoprotein exopolymer produced by *Pseudoalteromonas* sp. strain TG12. Appl. Environ. Microbiol..

[36] Drouillard S., Jeacomine I., Buon L., Boisset C., Courtois A., Thollas B., Morvan P.-Y., Vallée R., Helbert W. (2018). Structure of the exopolysaccharide secreted by a marine strain *Vibrio alginolyticus*. Mar. Drugs.

[37] Chalkiadakis E., Dufourcq R., Schmitt S., Brandily C., Kervarec N., Coatanea D., Amir H., Loubersac L., Chanteau S., Guezennec J. (2013). Partial characterization of an exopolysaccharide secreted by a marine bacterium, *Vibrio neocaledonicus* sp. nov., from New Caledonia. J. Appl. Microbiol..

[38] Roca C., Lehmann M., Torres C.A.V., Baptista S., Gaudêncio S.P., Freitas F., Reis M.A.M. (2016). Exopolysaccharide production by a marine *Pseudoalteromonas* sp. strain isolated from Madeira Archipelago ocean sediments. New Biotechnol..

[39] Mancuso Nichols C., Garon Lardière S., Bowman J.P., Nichols P.D., Gibson J.A.E., Guezennec J. (2005). Chemical characterization of exopolysaccharides from Antarctic marine bacteria. Microb. Ecol..

[40] Zhang Z., Cai R., Zhang W., Fu Y., Jiao N. (2017). A novel exopolysaccharide with metal adsorption capacity produced by a marine bacterium *Alteromonas* sp. JL2810. Mar. Drugs.

[41] Drouillard S., Jeacomine I., Buon L., Boisset C., Courtois A., Thollas B., Morvan P.-Y., Vallée R., Helbert W. (2015). Structure of an amino acid-decorated exopolysaccharide secreted by a *Vibrio alginolyticus* strain. Mar. Drugs.

[42] Atkins E.D., Sheehan J.K. (1971). The molecular structure of hyaluronic acid. Biochem. J..

[43] Carillo S., Casillo A., Pieretti G., Parrilli E., Sannino F., Bayer-Giraldi M., Cosconati S., Novellino E., Ewert M., Deming J.W. (2015). A unique capsular polysaccharide structure from the psychrophilic marine bacterium *Colwellia psychrerythraea* 34H that mimics antifreeze (glycol)proteins. J. Am. Chem. Soc..

[44] Moppert X., Le Costaouëc T., Raguénès G., Courtois A., Simon-Colin C., Crassous P., Costa B., Guezennec J. (2009). Investigations into the uptake of copper, iron and selenium by a highly sulphated bacterial exopolysaccharide isolated from microbial mats. J. Ind. Microbiol. Biotechnol..

[45] Decho A.W., Gutierrez T. (2017). Microbial extracellular polymeric substances (EPSs) in ocean systems. Front. Microbiol..

[46] Hall T. (1999). BioEdit: A user-friendly biological sequence alignement aditor and analysis program for Windows 95/98/NT. Nucleic Acids Symp. Ser..

[47] Lee H.G., Cowman M.K. (1994). An agarose-gel electrophoretic method for analysis of hyaluronan molecular-weight distribution. Anal. Biochem..

[48] Kamerling J.P., Gerwing G.J., Vliegenthart J.F., Clamp J.R. (1975). Characterization by gas-liquid chromatography-mass spectrometry and proton-magnetic-resonance spectroscopy of pertrimethylsilyl methyl glycosides obtained in the methanolysis of glycoproteins and glycopeptides. Biochem. J..

[49] Montreuil J., Bouquelet S., Debray H., Fournet B., Spik G., Strecker G., Chaplin M.F., Kennedy J.F. (1986). Glycoptoteins. Carbohydrate Analysis. A Pratical Approach.

[50] Chopin N., Sinquin C., Ratiskol J., Zykwinska A., Weiss P., Cerantola S., Le Bideau J., Colliec-Jouault S. (2015). A direct sulfation process of a marine polysaccharide in ionic liquid. BioMed Res. Int..

