# Supplementary material for: Deep-sea Hydrothermal Vent Bacteria as a Source of Glycosaminoglycan-Mimetic Exopolysaccharides
Source: Molecules. 2019 May 1;24(9):1703. doi: 10.3390/molecules24091703 (PMC6539532; doi:10.3390/molecules24091703)
Supplement: Supplementary file 1 [file molecules-24-01703-s001.pdf]

Figure S1. Example of electrophoretic analysis on agarose gel with Stains All staining performed on culture broth supernatants recovered after 48h of incubation of 21 deep-sea hydrothermal vent strains in Zobell-like medium supplemented with glucose during the screening step. GY785 EPS and HE800 EPS were used as references.

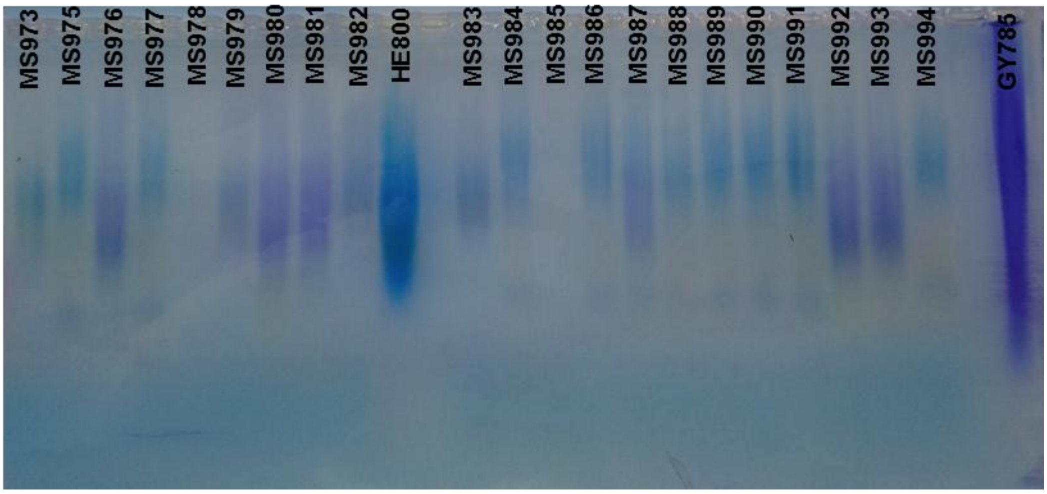

Table S1. Deep-sea hydrothermal vent strains selected for EPS production at laboratory scale.

Strain	Cruise	Cruise year	Cruise site	Sample location	EPS production
**BI6**	Biolau	1989	Southwestern Pacific Ocean (Lau Basin)	scratched shell of mussel	Yes
**BI15**	Biolau	1989	Southwestern Pacific Ocean (Lau Basin)	intervalvar water of mussel	Yes
**BI20**	Biolau	1989	Southwestern Pacific Ocean (Lau Basin)	sampling water at the base of a white smoker	Yes
**BI27**	Biolau	1989	Southwestern Pacific Ocean (Lau Basin)	hydrothermal fluid above a white smoker	Yes
**BI31**	Biolau	1989	Southwestern Pacific Ocean (Lau Basin)	sampling water at the way out of a black smoker	Yes
**BI54**	Biolau	1989	Southwestern Pacific Ocean (Lau Basin)	unknown	Yes
**BI77**	Biolau	1989	Southwestern Pacific Ocean (Lau Basin)	unknown	Yes
**BI93**	Biolau	1989	Southwestern Pacific Ocean (Lau Basin)	unknown	Yes
**BI112**	Biolau	1989	Southwestern Pacific Ocean (Lau Basin)	hydrothermal fluid above a white smoker	Yes
**BI121**	Biolau	1989	Southwestern Pacific Ocean (Lau Basin)	hydrothermal fluid in titane tube	Low amount
**BI134**	Biolau	1989	Southwestern Pacific Ocean (Lau Basin)	scratched shell of mussel	Yes
**BI145**	Biolau	1989	Southwestern Pacific Ocean (Lau Basin)	intervalvar water of mussel	Yes
**BI189**	Biolau	1989	Southwestern Pacific Ocean (Lau Basin)	crushed foot of Alviniconcha	Yes
**BI193**	Biolau	1989	Southwestern Pacific Ocean (Lau Basin)	crushed gills of Alviniconcha	Yes
**ST265**	Starmer	1989	Southwestern Pacific Ocean (North Fiji Basin)	intervalvar water of mussel	Low amount
**ST279**	Starmer	1989	Southwestern Pacific Ocean (North Fiji Basin)	crushed gills of Trochidae	Low amount
**ST285**	Starmer	1989	Southwestern Pacific Ocean (North Fiji Basin)	crushed digestive mass of Trochidae	Low amount
**ST308**	Starmer	1989	Southwestern Pacific Ocean (North Fiji Basin)	intervalvar water of mussel	Yes
**ST352**	Starmer	1989	Southwestern Pacific Ocean (North Fiji Basin)	crushed gills of Trochidae	Yes
**BI370**	Biolau	1989	Southwestern Pacific Ocean (Lau Basin)	crushed gills of Alviniconcha	Yes
**ST421**	Starmer	1989	Southwestern Pacific Ocean (North Fiji Basin)	intervalvar water of mussel	Yes
**ST422**	Starmer	1989	Southwestern Pacific Ocean (North Fiji Basin)	crushed digestive mass of mussel	Yes
**ST427**	Starmer	1989	Southwestern Pacific Ocean (North Fiji Basin)	crushed digestive mass of Alviniconcha	Yes
**BI724**	Biolau	1989	Southwestern Pacific Ocean (Lau Basin)	unknown	Yes
**AL749**	MMVT	1990	East Pacific Ridge (EPR 11°N, 13°N or 21°N)	unknown	Yes
**MA882**	Marvel	1993	Mid Atlantic Ridge (Snake Pit or Tag)	unknown	Yes
**MA886**	Marvel	1993	Mid Atlantic Ridge (Snake Pit or Tag)	shrimp	Low amount
**MA894**	Marvel	1993	Mid Atlantic Ridge (Snake Pit or Tag)	unknown	Yes
**MS911**	Microsmoke	1995	Mid Atlantic Ridge (Snake Pit)	colonization module	Yes
**MS937**	Microsmoke	1995	Mid Atlantic Ridge (Snake Pit)	colonization module	Yes
**MS944**	Microsmoke	1995	Mid Atlantic Ridge (Snake Pit)	colonization module	Yes
**MS950**	Microsmoke	1995	Mid Atlantic Ridge (Snake Pit)	colonization module	Yes
**MS969**	Microsmoke	1995	Mid Atlantic Ridge (Snake Pit)	colonization module	Yes
**AT1213**	Atos	2001	Mid Atlantic Ridge (Rainbow)	crushed Rimicaris	Yes
**AT1217**	Atos	2001	Mid Atlantic Ridge (Rainbow)	crushed Rimicaris	Low amount
**AT1219**	Atos	2001	Mid Atlantic Ridge (Rainbow)	active chimney	Yes
**PH1226**	Phare	2002	East Pacific Ridge (EPR 13°N)	unknown	Yes
**AT1240**	Atos	2001	Mid Atlantic Ridge (Rainbow)	crushed Rimicaris	Low amount

Table S2. Recovery yield, weight-average molecular weight (Mw), polydispersity index (Ip) and chemical composition (protein, sulfur and monosaccharide contents) of 31 EPS produced at laboratory scale. GY785 EPS and HE800 EPS were used as references.

**Strain**	**Yield** (g/L)	**Mw** (10^6 g/mol)	**Ip**	**Protein** (wt%)	**Sulfur** (wt%)	**Monosaccharide composition** (wt%)								
						**Rha**	**Fuc**	**Man**	**Gal**	**Glc**	**GlcA**	**GalA**	**GlcNAc**	**GalNAc**
**GY785**	0,55	3,6	1,05	15	3	0,7	0,3	1,8	17,6	22,9	9,5	4,9	0	0
**HE800**	0,35	1,8	1,5	1,9	0	0	0	0	0	0	17,1	0	26,2	8,4
**BI6**	0,47	2,6	1,03	3	0	0	0	0	0	0	6,9	18	11,2	4,1
**BI15**	0,68	2,5	1,05	3	0	0	0	0	0	0	10,4	7,6	11,6	11,2
**BI20**	0,58	2,3	1,03	6	0	0	0	0	0	0	4,1	18,7	13,3	1,6
**BI27**	0,57	2,5	1,04	3	0	0	0	0	0	0	10,8	7,8	11,5	12,4
**BI31**	0,27	3,2	1,2	5	0	0	0	0	0	0	6,1	11,6	8,2	2,9
**BI54**	0,42	2,5	1,03	5	0	0	0	0	0	0	11,6	8,6	8,3	7,7
**BI77**	0,32	2,4	1,04	5	0	0	0	0	0	0	7,6	8,2	9	12,6
**BI93**	0,42	2,4	1,02	6	0	0	0	0	0	0	8,4	7,9	9,6	13,6
**BI112**	0,34	2,7	1,07	5	0	0	0	0	0	0	2,3	10,9	7,1	0
**BI134**	0,6	1,4	1,1	4	0	0	0	0	0	0	8,9	6,6	11,4	18,3
**BI145**	0,49	2,4	1,03	4	0	0	0	0	0	0	7,8	6,1	8,5	14,7
**BI189**	0,6	2,3	1,04	4	0	0	0	0	0	0	5,6	3,2	6,1	0
**BI193**	0,84	2,3	1,1	4	0	0	0	0	0	0	8	5,7	11	9,5
**ST308**	1,03	2,3	1,04	3	0	0	0	0	0	0	0	18,1	8	0
**ST352**	0,67	3,0	1,05	4	0	0	0	0	0	0	0	17,5	6,5	0
**BI370**	1,25	2,8	1,03	4	0	0	0	0	0	0	0	19	4,2	0
**ST421**	0,83	2,5	1,2	12	2	2,3	2,2	0	13,9	21,8	6	1,7	0	0
**ST422**	0,7	2,6	1,07	1	0	0	0	0	0	0	24,3	0	21,4	16,5
**ST427**	0,76	2,4	1,02	2	0	0	0	0	0	0	21,9	0	19,4	15,8
**BI724**	0,58	2,2	1,03	9	0	0	0	3,1	2,3	6,6	5,6	1,8	7,4	1,7
**AL749**	0,68	3,0	1,01	6	0	0	0	0	2,5	5,8	2,8	0	9,7	6,4
**MA882**	0,12	5,1	1,05	6	0	0	0	0	0	0	7,1	1,1	6,9	3,2
**MA894**	0,38	2,8	1,04	6	0	0	0	0	0	0	10,6	8,8	11,1	14,8
**MS911**	0,3	3,0	1,05	4	0	0	0	0	0	0	3,6	13,6	12,6	3
**MS937**	0,4	3,1	1,02	4	0	0	0	0	0	0	18,5	3,3	18,3	13,1
**MS944**	0,45	1,9	1,1	4	0	0	0	0	0	0	3,4	17,2	11,5	4,3
**MS950**	0,92	3,0	1,03	16	0	0	0	0	5,9	11	6,4	1,8	4,5	1,6
**MS969**	0,26	2,8	1,24	13	3	11,1	0	0	1,8	15,9	5,6	0	0	0
**AT1213**	0,77	2,1	1,09	4	0	0	0	0	10,6	2	9,3	5,3	10,3	3,1
**AT1219**	0,44	3,2	1,16	7	0	0	0	0	12,8	5,4	13	6,1	10,5	17,6
**PH1226**	0,29	2,2	1,06	5	0	0	0	0	12,8	4,8	11,6	5,8	10,5	14,8
